# Superior vena cava isolation with pulsed field ablation in the presence of defibrillator leads

**DOI:** 10.1002/joa3.12848

**Published:** 2023-04-18

**Authors:** Dimitrios Tsiachris, Ioannis Doundoulakis, Athanasios Kordalis, Christos‐Konstantinos Antoniou, Konstantinos Tsioufis

**Affiliations:** ^1^ First Department of Cardiology “Hippokration” Hospital, National and Kapodistrian University Athens Greece; ^2^ Athens Heart Center Athens Medical Center Athens Greece

**Keywords:** atrial fibrillation, defibrillator, pulsed field ablation

Two patients with a history of dilated cardiomyopathy, dual chamber defibrillator implantation, and worsening heart failure because of persistent atrial fibrillation (AF) underwent catheter ablation of AF. Pulsed field ablation (PFA) was selected for the index procedure in these decompensated heart failure patients with enlarged atria.^1^

The 12‐Fr over‐the‐wire pentaspline PFA catheter (Farawave) was advanced through a 13‐Fr steerable sheath (Faradrive) to the ostium of each PV, and a total of eight PFA lesions were applied per vein. Phrenic nerve function was evaluated by direct phrenic capture and by observing diaphragmatic motion during inspiration. For posterior wall ablation, the catheter was placed into a flower configuration and positioned along the posterior left atrium to deliver overlapping sets of pulses at each location. As per institution protocol, superior vena cava (SVC), as a potential nonpulmonary trigger for AF, is isolated when active despite a risk of sinus node injury.^2^

Basket configuration (Figure 1) and two sets of two lesions at the trunk of the vessel (with rotation between each pair) were used for SVC isolation in the presence of atrial and defibrillator leads, without any impact on device and leads measurements or sinus node function (Figure 2). We observed incidence of phrenic capture during PFA, and no evidence of phrenic injury was detected after the procedure. Both patients were in AF and cardioverted into sinus rhythm post‐SVC isolation. They remained at sinus rhythm at regular 3‐month follow‐up while device interrogation revealed intact sinus node function.

**FIGURE 1:**
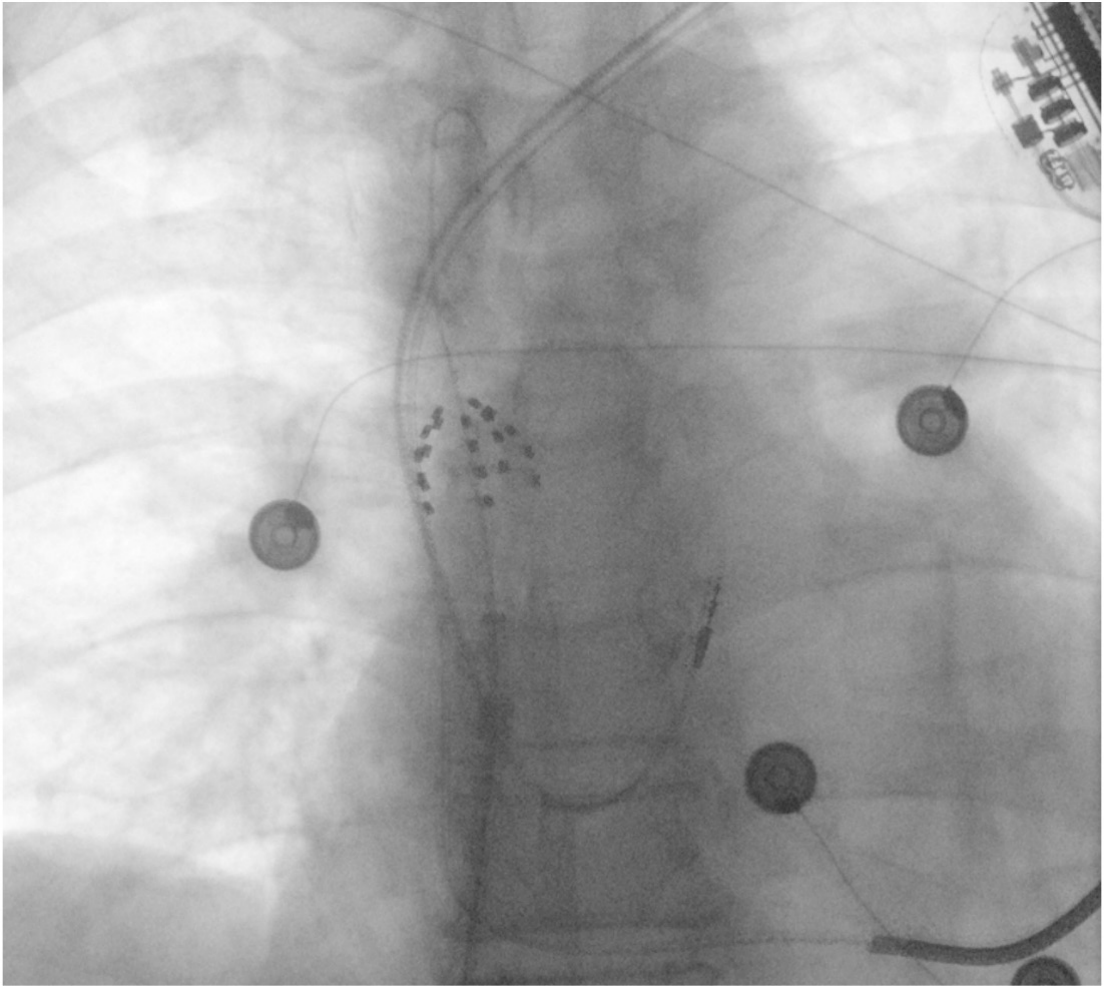
Basket configuration of Farapulse catheter in the superior vena cava in the presence of defibrillator leads.

**FIGURE 2:**
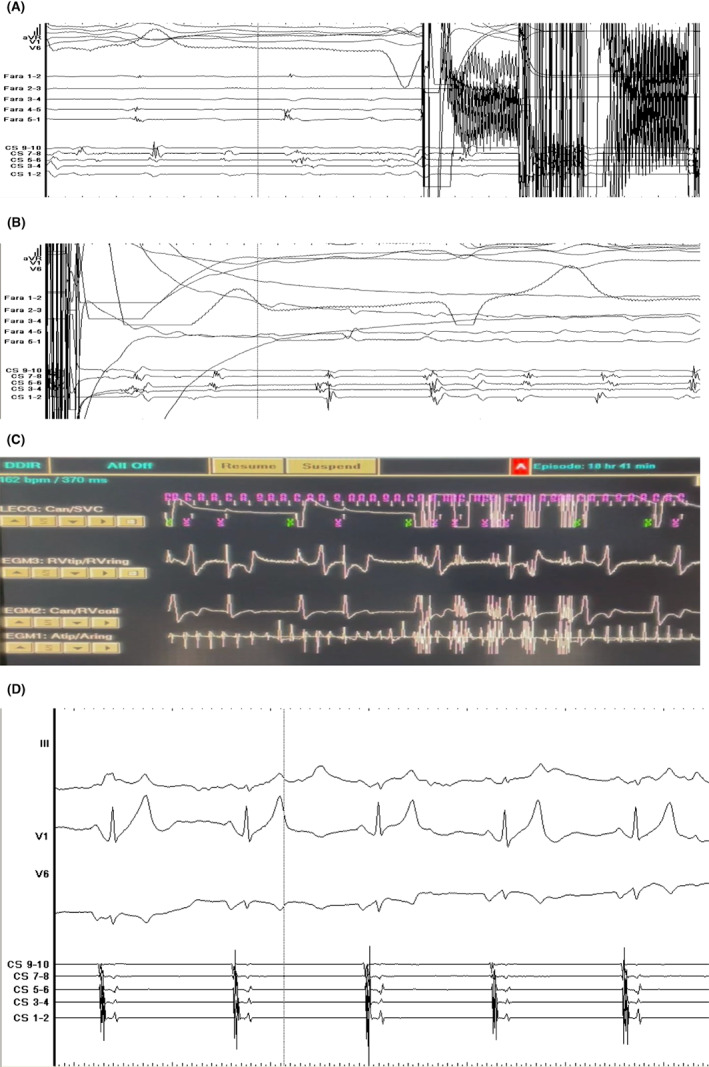
(A) Superior vena cava potentials in Farapulse (Fara 1–5) prior the first pulsed field ablation application. (B) Superior vena cava isolation in Farapulse (Fara 1–5) after the first pulsed field ablation application. (C) Real‐time defibrillator interrogation depicting noise during pulsed field ablation application (all therapies were set off). (D) Sinus rhythm preservation post superior vena cava isolation.

Only 4.2% of operators in the MANIFEST‐PF multinational survey (including 1758 consecutive unselected AF patients) performed SVC isolation by mean of PFA occasionally.^3^ This is the first report of off‐label pulsed field ablation‐mediated SVC isolation without device or lead malfunction.

Human studies evaluating cardiac implantable electrical devices during ablation procedure remain limited. A recent study by a high‐volume center indicated that PFA is a safe and feasible method in patients with devices in the terms of alterations in pacing, sensing, or impedance parameters, inappropriate anti‐tachycardia pacing, and ICD shock and leads dislodgment.^4^ Indeed, our pilot procedures with PFA applications in the SVC of AF patients with implanted devices confirmed the previous results without apparent additional risk because of energy application adjacent to the electrodes.

## FUNDING INFORMATION

None.

## CONFLICT OF INTEREST STATEMENT

The authors declare no conflict of interests for this article.

## DECLARATIONS

**Approval of the research protocol:** N/A.

**Informed Consent**: Ν/Α.

**Registry and the Registration No. of the study/trial:** N/A.

**Animal Studies:** N/A.

## Data Availability

Data are available in the article.
